# Atropine does not prevent hypoxemia and bradycardia in tracheal intubation in the pediatric emergency department: observational study

**DOI:** 10.1590/1984-0462/2024/42/2022220

**Published:** 2023-11-03

**Authors:** Vitor Emanoel de Lemos Carvalho, Thomaz Bittencourt Couto, Bruno Marcelo Herculano Moura, Cláudio Schvartsman, Amélia Gorete Reis

**Affiliations:** a Universidade de São Paulo – São Paulo, SP, Brazil.

**Keywords:** Atropine, Hypoxemia, Bradycardia, Fatty acid desaturases, Intubation, Atropina, Hipoxemia, Bradicardia, Ácidos graxos dessaturases, Intubação

## Abstract

**Objective:**

The benefit of atropine in pediatric tracheal intubation is not well established. The objective of this study was to evaluate the effect of atropine on the incidence of hypoxemia and bradycardia during tracheal intubations in the pediatric emergency department.

**Methods:**

This is a single-center observational study in a tertiary pediatric emergency department. Data were collected on all tracheal intubations in patients from 31 days to incomplete 20 years old, performed between January 2016 and September 2020. Procedures were divided into two groups according to the use or not of atropine as a premedication during intubation. Records with missing data, patients with cardiorespiratory arrest, cyanotic congenital heart diseases, and those with chronic lung diseases with baseline hypoxemia were excluded. The primary outcome was hypoxemia (peripheral oxygen saturation ≤88%), while the secondary outcomes were bradycardia (decrease in heart rate >20% between the maximum and minimum values) and critical bradycardia (heart rate <60 bpm) during intubation procedure.

**Results:**

A total of 151 tracheal intubations were identified during the study period, of which 126 were eligible. Of those, 77% had complex, chronic underlying diseases. Atropine was administered to 43 (34.1%) patients and was associated with greater odds of hypoxemia in univariable analysis (OR: 2.62; 95%CI 1.15–6.16; p=0.027) but not in multivariable analysis (OR: 2.07; 95%CI 0.42–10.32; p=0.37). Critical bradycardia occurred in only three patients, being two in the atropine group (p=0.26). Bradycardia was analyzed in only 42 procedures. Atropine use was associated with higher odds of bradycardia in multivariable analysis (OR: 11.00; 95%CI 1.3–92.8; p=0.028).

**Conclusions:**

Atropine as a premedication in tracheal intubation did not prevent the occurrence of hypoxemia or bradycardia during intubation procedures in pediatric emergency.

## RESUMO

**Objetivo:**

Avaliar o efeito da atropina na incidência de hipoxemia e bradicardia durante a intubação orotraqueal no departamento de emergência pediátrica.

**Métodos:**

Estudo observacional, realizado em departamento de emergência pediátrica terciário em que foram analisados os registros de intubações orotraqueais de pacientes com 31 dias a 20 anos incompletos, entre janeiro de 2016 e setembro de 2020. Os procedimentos foram divididos em dois grupos de acordo com o uso ou não da atropina como pré-medicação durante a intubação. Foram excluídos os procedimentos com falhas no preenchimento dos dados, pacientes com parada cardiorrespiratória, cardiopatias congênitas cianóticas, e aqueles com pneumopatias crônicas com hipoxemia basal. O desfecho primário foi hipoxemia (saturação periférica de oxigênio ≤88%), enquanto os desfechos secundários foram bradicardia (queda >20% entre a frequência cardíaca máxima e mínima) e bradicardia crítica (frequência cardíaca <60 bpm) durante o procedimento de intubação

**Resultados:**

Foram identificados 151 procedimentos de intubação orotraqueal, sendo 126 elegíveis para o estudo. Desses, 77% tinham doenças subjacentes complexas e crônicas. A atropina foi administrada em 43 (34,1%) pacientes e foi associada a maiores chances de hipoxemia na análise univariada (OR: 2,62; IC95% 1,15–6,16; p=0,027), porém, não na análise multivariada (OR: 2,073; IC95% 0,416–10,32; p=0,373). A bradicardia crítica ocorreu em apenas três pacientes, sendo dois no grupo atropina (p=0,268). A bradicardia foi analisada em apenas 42 procedimentos. O uso de atropina foi associado a maior probabilidade de bradicardia (OR: 11,00; IC95% 1,3–92,8; p=0,028) na análise multivariável.

**Conclusões:**

Atropina como pré-medicação na intubação orotraqueal não evitou a ocorrência de hipoxemia ou bradicardia durante os procedimentos de intubação na emergência pediátrica.

## INTRODUCTION

Tracheal intubation (TI) is a critical step in childcare in the emergency room and can cause deleterious effects such as hypoxemia, hypotension, bradycardia, and death,^1^ especially in critically ill patients.

Children's anatomical particularities make laryngoscopy challenging and intubation more likely to fail when compared to adults, due to their large occiput, narrow larynx, and a more anterior and elliptical epiglottis. Hypoxemia is one of the most common adverse events during pediatric TI.^2,3^

Several efforts and extensive research were carried out seeking for strategies to reduce potential risks associated with this procedure, including the universal recommendation of rapid sequence intubation (RSI). RSI is the method of choice to prevent complications during intubation. It is a systematic approach that encompasses pre-oxygenation, premedication, and the use of sedatives and neuromuscular blockers (NMB)^4,5^ to facilitate the correct tube placement.

The use of atropine, an anticholinergic drug, as premedication for RSI, is controversial. It was initially recommended before TI to avoid reflex bradycardia during laryngoscopy,^6^ as a result of vagal and glossopharyngeal nerve^7^ activity.

Recent studies have shown no benefit from the use of atropine in RSI for the outcomes of bradycardia and hypoxemia during TI.^8-10^ Thus, after careful analysis, the main guidelines in advanced life support do not recommend the routine use of atropine before intubations in the emergency department.^11-13^ However, in clinical practice, atropine is still often used in these procedures.^14^

The benefit of administering atropine during TI in children is not well established in the literature. Further investigations are essential to analyze the role of atropine in reducing complications during TI.

The study objective was to test the association of atropine use with the occurrence of hypoxemia and bradycardia during pediatric TI.

## METHOD

This was a retrospective observational study at the emergency department of the Children's Institute of Clinics Hospital of the Faculty of Medicine, University of São Paulo (ICr-HCFMUSP), which is a university-affiliated tertiary pediatric hospital. Patients aged 31 days to 19 years who underwent TI from January 2016 to September 2020 were eligible for the study.

For data collection, an institutional database of all TI, complementary to patient's medical records, was adopted. The institutional database was constructed before this work started using the variables recommended by the international Near4kids registry:^15^ age, weight, gender, use of noninvasive ventilation (NIV) prior to the TI procedure, indication for TI, professional who performed TI, difficult airway assessment, medications used, TI method, adverse events, and clinical outcomes.

The inclusion criteria were records containing at least age, weight, TI indication, number of TI attempts, drugs for use in the RSI, TI device, and adverse events. The study excluded patients with cardiorespiratory arrest, cyanogenic heart disease, and severe pneumopathy, as well as records with missing data.

The procedures were divided into two groups: atropine and non-atropine during TI.

The Pediatric Early Warning Score (PEWS)^16^ was applied for severity comparison between groups as shown in Table 1. This score is graduated up to 11 points, with five or more points associated with a high risk of clinical deterioration and the need for immediate medical intervention.

**Table 1:** Comparison of baseline variables, procedure indications and characteristics, and adverse events between the two groups.

Variables	Category	Atropine		p-value
		No (n=83, %)	Yes (n=43; %)	
Age (years)	1 month to 1	22 (26.5)	29 (67.4)	<0.001
	1–8	45 (54.2)	11 (25.6)	
	> 8	16 (19.3)	3 (7)	
Age (months)	Mean (SD)	50.1 (53.4)	23.6 (45.2)	0.004
Weight (kg)	Mean (SD)	16.2 (13.8)	9.3 (10.8)	0.003
Sex	Male	42 (50.6)	27 (62.8)	0.260
PEWS	Mean (SD)	6.4 (1.2)	6.1 (1.3)	0.260
TI pre-ventilation devices				
NIV/CPAP/HFNC		21 (26.6)	23 (56.1)	0.003
TI indications				
Oxygenation failure		33 (39.8)	23 (53.5)	0.200
Procedure performance		3 (3.6)	0 (0)	0.550
Ventilation failure		15 (18.)1	12 (27.9)	0.295
Apnea and bradycardia		10 (12)	8 (18.6)	0.466
Upper airways obstruction		2 (2.4)	1 (2.3)	0.999
Neuromuscular weakness		2 (2.4)	1 (2.3)	0.999
Hemodynamic instability		25 (30.1)	11 (25.6)	0.744
Absence of protective airway reflexes		15 (18.1)	3 (7)	0.156
TI procedure variables				
Professional who performed the intubation	Second-year pediatric residents	38 (45.8)	17 (39.5)	0.535
	Third-year pediatric residents	16 (19.3)	12 (27.9)	
	Emergency pediatrician or anesthetist	29 (34.9)	14 (32.6)	
Difficulty of ventilation with BMV		17 (21.5)	9 (23.1)	0.999
Medications used in TI				
NMB in RSI	None	12 (14.5)	3 (7)	0.348
	Rocuronium	71 (85.5)	40 (93)	
Sedative in RSI	None	3 (3.6)	0 (0)	0.104
	Ketamine	19 (22.9)	20 (46.5)	
	Midazolam	9 (10.8)	3 (7)	
	Propofol	3 (3.6)	0 (0)	
	Midazolam + ketamine	39 (47)	16 (37.2)	
	Midazolam + propofol	1 (1.2)	0 (0)	
	Midazolam + propofol + ketamine	1 (1.2)	2 (4.7)	
	Others	8 (9.6)	2 (4.7)	
During TI				
TI device	Videolaryngoscopy	62 (74.7)	18 (41.9)	<0.001
	Direct laryngoscopy	18 (21.7)	23 (53.5)	
TI device	Others	3 (3.6)	2 (4.7)	<0.001
Glottic exposure at TI	I or II	79 (95.2)	36 (83.7)	0.045
	III or IV	4 (4.8)	7 (16.3)	
TI attempt	Mean (SD)	2.55 (1.93)	3.42 (2.33)	0.028
TI adverse events				
None		46 (55.4)	21 (48.8)	0.607
Death		3 (3.6)	0 (0)	0.550
Cardiopulmonary arrest		4 (4.8)	0 (0)	0.298
Selective bronchial intubation		8 (9.6)	3 (7)	0.748
Esophageal TI		12 (14.5)	9 (20.9)	0.501
Hypotension		0 (0)	1 (2.3)	0.341
Lip trauma		1 (1.2)	0 (0)	0.999
Vomiting		5 (6)	0 (0)	0.165
Pneumothorax		0 (0)	1 (2.3)	0.341
Arrhythmia (including bradycardia <60 bpm)		1 (1.2)	2 (4.7)	0.268
Successful TI approach		80 (96.4)	40 (93)	0.410

TI: tracheal intubation; BMV: bag-mask ventilation; PEWS: pediatric early warning score; NIV: noninvasive ventilation; CPAP: continuous positive airway pressure; HFNC: high flow nasal cannula; RSI: rapid sequence intubation; NMB: neuromuscular blocker; SD: standard deviation; Min: minimum; Max: maximum.

The patients were categorized according to age as: 1 month to 1 year, 1–8 years, and >8 years, due to the particularities of the airways of each age group that influence laryngoscopy.^2^

The main outcomes, hypoxemia and bradycardia, were assessed in both groups. The standard dose of atropine was 0.02 mg/kg, with a minimum of 0.1 mg and a maximum of 1 mg.^6^

The primary outcome, hypoxemia, was defined as a peripheral oxygen saturation (SpO_2_) ≤88% throughout TI until the beginning of ventilation. The secondary outcomes were bradycardia, defined as a decrease in heart rate (HR) >20% between the maximum and minimum (max and min HR) values, and critical bradycardia, defined as a HR <60 beats per minute (bpm). The maximum HR was recorded immediately before the procedure, and the minimum was recorded as the lowest value throughout the procedure until the beginning of ventilation. Critical bradycardia was examined in all intubations, while bradycardia was examined in only 42 procedures.

Oxygenation failure was considered in cases of severe respiratory distress associated with hypoxemia that was unresponsive to supplemental oxygen. Ventilation failure was considered when there were signs of respiratory failure, such as severe distress and muscle fatigue. Although there are blood gas parameters for oxygenation and ventilation failures, herein we adopted the clinical criteria.

The project was approved by the hospital's Research Ethics Board (REB), under number 96090418.2.0000.0068, from April 10, 2018. For this work, the need for informed consent was waived by the REB.

Fisher's exact test or chi-square test was used to assess possible differences between groups on qualitative variables such as hypoxemia and bradycardia. Comparisons of quantitative variables between the two independent groups were performed with the nonparametric Mann-Whitney U test or the parametric Student's *t*-test. The Shapiro-Wilk test was used to assess the normality of data in each group. When the assumption of normality was not met or the number of patients in a given group was less than ten, the nonparametric Mann-Whitney U test was applied.

Subsequently, an univariable logistic regression model was fitted to assess the factors associated with the outcomes — the measure of association was given by the odds ratio (OR) with its respective 95% confidence interval (CI). Furthermore, a multivariable logistic regression model was fitted as a function of the atropine use adjusting by clinically important factors and confounders, such as age, NIV, continuous positive airway pressure (CPAP), high flow nasal cannula (HFNC), professional who performed TI, difficult ventilation with bag-mask ventilation (BMV), maximum mouth opening, NMB in RSI, TI device, PEWS, and TI attempts. All hypotheses were two-sided and tested at a 5% significance level. Statistical analyses were performed using R version 4.12 (R Core Team, 2020). The sample size calculation was based on data from a previous study in which hypoxemia was observed in 47% of TI.^17^ The sample size necessary to give the research 80% power to find differences ≥50% between groups should be approximately 80 patients per group (GraphPad Statmate 1.01).

## RESULTS

**Figure 1:**
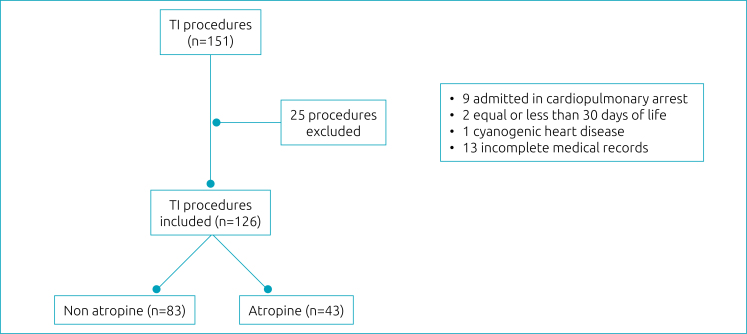
Flowchart of procedures enrolled.

There were 151 TI procedures during the study period, 126 of which were eligible, as shown in Figure 1, with atropine applied to 43 (34.1%) patients. In all intubations, the option to use atropine was defined by the staff during the procedures, without the researchers’ awareness. The data were recorded by physicians involved in the processes.

RSI was performed in 88.1% of cases and rocuronium was the NMB of choice in 100% of cases. The main indication for TI was oxygenation failure (44.4%), followed by hemodynamic instability (28.5%), and ventilation failure (21.4%). A second-year pediatric resident performed 43.7% of the TI. A videolaryngoscope (McGrath Mac) was used in 63.5% of the procedures. Adverse events occurred more frequently in patients aged between 1–8 years (44%). In this study, 77% of the patients had complex underlying diseases and only one procedure was indicated due to trauma (severe traumatic brain injury).

In the case of a failed first TI attempt or severe clinical instability prior to the procedure, intubation was performed by more experienced pediatric emergency physicians (30.9%) or anesthetists (0.03%).

Table 1 shows the comparison of baseline data, procedure characteristics, and adverse events between the two groups. It was observed that the procedures in which atropine was used (43) had a higher number of intubation attempts than those who did not use atropine (83) for RSI (3.42 versus 2.55, respectively; p-value[p]=0.02). Clinical severity was similar between groups according to the criterion PEWS applied, with a median score of six for both groups (p=0.26).

Hypoxemia occurred in 83 (65%) procedures, 34 (79.1%) in the atropine group and 49 (59%) in the non-atropine group, with statistical significance between them (p=0.04) (Table 2).

**Table 2:** Group comparison according to occurrence of hypoxemia and heart rate decreasing.

Variable		Atropine		p-value
		No n (%) n=83	Yes n (%) n=43	
Hypoxemia*		49 (59.0)	34 (79.1)	0.040
Bradycardia†		3 (10.7)	5 (35.7)	0.092
Lower HR	Pre-medication	3 (11.5)	4 (33.3)	0.450
	Ventilation/Oxygenation	3 (11.5)	1 (8.3)	
	During sedative use	6 (23.1)	1 (8.3)	
	During NMB use	1 (3.8)	1 (8.3)	
	During TI passage/check	13 (50)	5 (41.7)	

HR: heart rate; NMB: neuromuscular blocker; TI: tracheal intubation.

* peripheral oxygen saturation (SpO_2_)≤ 88%;

† any decrease >20% between maximum and minimum heart rate.

In the univariable analysis, the use of atropine was related to a 2.6-fold increase in hypoxemia odds (OR: 2.62; 95%CI 1.11–6.16; p=0.02). The variables: age, PEWS, professional who performed TI, successive TI attempts, and the use of videolaryngoscope for intubation were also statistically associated with hypoxemia. In the multiple analysis, atropine use was not associated with hypoxemia odds (OR: 2.07; 95%CI 0.41–10.32; p=0.37), as shown in Table 3. For this outcome, the variables selected in the stepwise regression and included in the model were difficult BMV (OR: 5.64; 95%CIC 1.16–27.45; p=0.032), clinical severity measured by the PEWS score (OR: 3.156; 95%CI 1.51– 6.56; p=0.002), and successive attempts of TI (OR: 1.91; 95%CI 1.13–3.21; p=0.015).

**Table 3:** Univariable and multivariable logistic regression analysis of hypoxemia (peripheral oxygen saturation ≤88%) during tracheal intubation.

Variable	Category	Univariable logistic regression model				Multivariable logistic regression model			
		OR	95%CI		p-value	OR	95%CI		p-value
			Lower	Upper			Lower	Upper	
Use of atropine	No	Ref				Ref			
	Yes	2.621	1.115	6.164	0.027	2.073	0.416	10.321	0.373
Age (months)	1-unit increment	0.990	0.983	0.997	0.008	0.998	0.986	1.009	0.685
NIV/CPAP/HFNC	No	Ref				Ref			
	Yes	1.555	0.703	3.442	0.276	1.814	0.457	7.196	0.397
Professional who performed the intubation	Second-year pediatric residents	Ref				Ref			
	Third-year pediatric residents	2.593	0.977	6.877	0.056	0.780	0.151	4.033	0.767
	Others	5.333	2.028	14.026	0.001	2.661	0.487	14.555	0.259
Difficult ventilation with BMV	No	Ref				Ref			
	Yes	1.864	0.681	5.103	0.225	5.645	1.160	27.459	0.032
Maximum mouth opening	No	Ref				Ref			
	Yes	2.000	0.899	4.452	0.090	1.836	0.465	7.257	0.386
NMB in RSI	No	Ref				Ref			
	Rocuronium	1.333	0.441	4.029	0.610	1.902	0.324	11.17	0.477
TI device	Direct laryngoscopy					Ref			
	Videolaryngoscopy	0.329	0.140	0.772	0.011	1.335	0.307	5.811	0.700
PEWS	1-unit increment	1.454	1.019	2.074	0.039	3.156	1.516	6.568	0.002
TI attempts	1-unit increment	1.860	1.376	2.514	<0.001	1.911	1.136	3.213	0.015

BMV: bag-mask ventilation; OR: odds ratio; CI: confidence interval; PEWS: Pediatric Early Warning Score; TI: tracheal intubation; NMB: neuromuscular blocker; NIV: noninvasive ventilation; CPAP: continuous positive airway pressure; HFNC: high flow nasal cannula; RSI: rapid sequence intubation.

Regarding the secondary outcome, critical bradycardia, three occurrences were found, two in the atropine group and one in the non-atropine group, and these small numbers did not allow any reliable statistical analysis.

Bradycardia was assessed in only 42 procedures, and occurred in five of 14 records in the atropine group and in three of 28 in the non-atropine group, with no statistical difference between them (p=0.09) and in the univariable analysis (OR: 4.63; 95%CI 0.91–23.42; p=0.06). However, in the multiple model, atropine use was associated with greater odds of bradycardia, compared to procedures in which atropine was not used (OR: 11.05; 95%CI 1.30–92.89; p=0.02), as shown in Table 4.

**Table 4:** Multivariable analysis of bradycardia (>20% decrease in heart rate) during tracheal intubation.

Variable	Category	OR	95%CI		p-value
			Lower	Upper	
Use of atropine	No	Ref			
	Yes	11.005	1.304	92.891	0.028
Age (months)	Continuous	1.010	0.993	1.028	0.236
TI device	Direct laryngoscopy Videolaryngoscopy	Ref			
		2.546	0.197	32.830	0.474
Constant					0.007

OR: odds ratio; CI: confidence interval; TI: tracheal intubation.

## DISCUSSION

This observational study showed that atropine use during TI in a tertiary pediatric emergency department was significantly associated with great odds of hypoxemia in univariable analysis, but not in multivariable analysis. Atropine use was also associated with increased odds of bradycardia, but only in the multivariable analysis. Critical bradycardia during TI was a rare event.

Atropine was used in 34.1% of the TI, and this rate was within the range reported by other published studies (24.2 to 47.5%).^8,9,18^ However, contrary to what is believed, the use of atropine during RSI in the study population did not protect against the selected outcomes.

The primary outcome, hypoxemia, occurred in 65% of the procedures. The choice of the cutoff SpO_2_≤88% aimed at increasing sensitivity to detect early deterioration which explains the high frequency of hypoxemia we observed. Published data reported lower incidences of hypoxemia during TI, ranging from 4 to 33%, but these studies adopted different cutoffs for oxygen saturation, making it difficult to compare the results.^19-21^ In addition, some studies included patients with different characteristics, such as trauma victims, which may have greater oxygen reserves and, consequently, less frequent desaturations during TI.^19-21^ It is important to notice that the population included in the current study was mostly patients with complex chronic diseases (77%), with acute life-threatening complications. These patients often require frequent follow-up care with specialists and a multidisciplinary outpatient team and are at higher risk of adverse events during TI.

This current work demonstrated a higher incidence of hypoxemia in the atropine group (79.1%) than in the non-atropine group (59%); this trend was also reported by a similar study by Fastle et al.^8^ Both were performed in a pediatric emergency setting, with respiratory diseases being the main indications for TI. In addition, the mean age of patients who received atropine was also similar (23.6 vs. 22.5 months, respectively).

Factors independently associated with the occurrence of hypoxemia in the present study were: difficulty in bag-mask ventilation, the severity of clinical condition, and successive TI attempts. Although the use of atropine was associated with episodes of hypoxemia in the comparative analysis between groups and in the univariable analysis, this relationship was not observed in the multivariable analysis. The results indicated that the patient's severity prior to intubation and the difficulties encountered during the laryngoscopy were determining factors for hypoxemia and bradycardia, making it difficult to interpret the independent role of atropine.

Successive TI attempts are associated with the occurrence of hypoxemia^22-24^ and, therefore, this factor should be considered in the context of atropine use. In the present study, the mean number of TI attempts was 2.85 in the total population, higher in the atropine group than in the non-atropine group (3.42 vs. 2.55, respectively). Lee et al.^22^ also reported a higher use of vagolytic drugs in patients who required three or more TI attempts. It is not evident that atropine can prevent hypoxemia, mainly considering that other factors may lead to that adverse effect.

Atropine does not seem to be the best way to avoid hypoxemia during the TI procedure, and it is essential to prioritize efforts in hemodynamic stabilization of the critically ill patient and ensure adequate ventilation and oxygenation. It is not certain, as well, that the deleterious effect of multiple laryngoscopy attempts during RSI is mitigated when atropine is administered as a premedication, making it a doubtful indication.

Currently, the use of videolaryngoscopes in pediatric intubation has gained attention since it allows for better visualization of the epiglottis and facilitates securing an advanced airway even in patients with a difficult airway.^1,25^ Thus, it is essential to adjust for the use of videolaryngoscope, as showed in Table 3, in the analysis of the effect of atropine on the outcomes of hypoxemia and bradycardia. In this study, the use of videolaryngoscope was associated with a lower chance of hypoxemia, but only in the univariable analysis. Some confounding factors must be considered in this interpretation; patients younger than one year were preferably intubated with direct laryngoscopy, received atropine as premedication more often, and presented desaturation more frequently.

Critical bradycardia was rare, apparently with no difference between the two groups. A similar result was observed by Kovacich et al.,^9^ in a pediatric population that included neonatal patients and whose main indication for intubation was trauma. Even with different methodologies, the use of atropine did not appear to prevent bradycardia in both studies.

Other studies also reported a low incidence of critical bradycardia, such as those by Li et al.^10^ (0.8%) and Jones et al.^18^ (0.06%). This low incidence can be explained by the choice of a very low HR value to define critical bradycardia, which is not frequently observed during TI in the pediatric population, except in patients at imminent risk of cardiorespiratory arrest. Although an HR <60 bpm can be a low discriminatory value, its assessment is crucial, since bradycardia is a late response to persistent hypoxemia; thus, hypoxemia seems to be a more sensitive parameter to detect a patient's clinical deterioration during the procedure.^26^

Jones et al.^18^ advocated for atropine use during TI in their research in an intensive care unit with 327 patients and observed a lower incidence of arrhythmias in the atropine group (4.5%) vs non-atropine group (26,5%,) but bradycardia was not analyzed. Another peculiarity is that they included neonates in the population. These differences in study designs require caution in comparisons with current results.

Factors that may influence bradycardia during TI, besides atropine, need to be considered. Although succinylcholine has been pointed out in other studies as a factor associated with bradycardia,^27,28^ this could not be analyzed in the current work since rocuronium was the only NMB used in patients. This observation is in line with the contemporary literature^9,15^.

Desalu et al.^29^ investigated the hemodynamic effects of atropine during TI and adopted the same criteria for bradycardia that we used (a decrease >20% between max and min HR). However, bradycardia was not observed in any patient, which can be explained by the fact that only those undergoing elective anesthesia and without acute diseases were considered, a population quite different from the current study.

The findings reported herein show that although atropine has potential to prevent bradycardia, acting in the sinoatrial node, it seems not effective in preventing bradycardia during TI procedures. The studies that advocate its use provide weak scientific evidence since they were carried out in a setting outside the emergency department and did not include critically ill patients. Data from this study suggest that the use of atropine during TI in critically ill patients does not prevent bradycardia and hypoxemia.

The present work has some limitations, once it is observational and conducted in a single center with the already known biases related to its design. Observational studies have a lower evidence standard than the experimental methods, are more prone to bias and confounding, and cannot be used to demonstrate causality. On the other hand, it has good internal validity because it was held in a specific population of patients with complex chronic diseases in emergency situations, certainly adding important information about clinical practice. Conducting randomized, double-blind clinical trials is not easy, and is often unfeasible in intubations in emergency departments. Thus, observational studies are of great value in the context of pediatric emergencies. Furthermore, the study did not achieve the calculated minimum sample size because of the reduction in the number of consultations during the Covid-19 pandemic. The small number of participants explains the wide CI for the OR of bradycardia in the atropine group. Even with those limitations, the hypothesis that atropine is not associated with a reduction in the incidence of hypoxemia and bradycardia during TI is not compromised since the incidence of those adverse effects was higher in the atropine group.

In conclusion, in this study, atropine use did not decrease the incidence of hypoxemia and bradycardia during TI in children in the pediatric emergency department.
